# Cancer-mitochondria-targeted photodynamic therapy with supramolecular assembly of HA and a water soluble NIR cyanine dye†
†Electronic supplementary information (ESI) available. See DOI: 10.1039/c7sc03169f


**DOI:** 10.1039/c7sc03169f

**Published:** 2017-10-13

**Authors:** Ajesh P. Thomas, L. Palanikumar, M. T. Jeena, Kibeom Kim, Ja-Hyoung Ryu

**Affiliations:** a Department of Chemistry , School of Natural Sciences , Ulsan National Institute of Science and Technology (UNIST) , Ulsan-44919 , South Korea . Email: jhryu@unist.ac.kr

## Abstract

Mitochondria-targeted cancer therapies have proven to be more effective than other similar non-targeting techniques, especially in photodynamic therapy (PDT). Indocyanine dye derivatives, particularly IR-780, are widely known for their PDT utility. However, poor water solubility, dark toxicity, and photobleaching are limiting factors for these dyes, which otherwise show promise based on their good absorption in the near-infrared (NIR) region and mitochondria targeting ability. Herein, we introduce an indocyanine derivative (**IR-Pyr**) that is highly water soluble, exhibiting higher mitochondrial targetability and better photostability than IR-780. Furthermore, electrostatic interactions between the positively charged **IR-Pyr** and negatively charged hyaluronic acid (HA) were utilized to construct a micellar aggregate that is selective towards cancer cells. The cancer mitochondria-targeted strategy confirms high PDT efficacy as proved by *in vitro* and *in vivo* experiments.

## Abstract 2

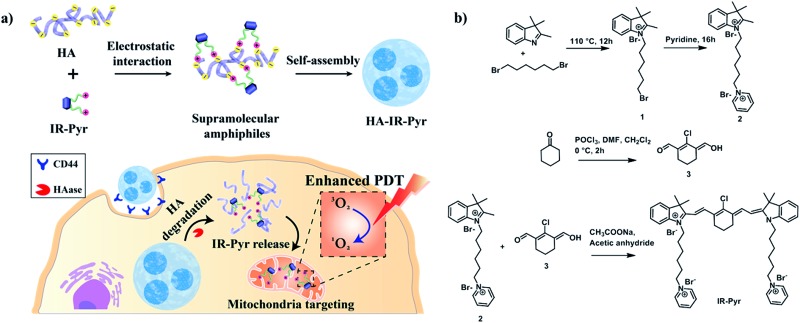
Herein, we introduce an indocyanine derivative (**IR-Pyr**) that is highly water soluble, exhibiting higher mitochondrial targetability and better photostability than IR-780.

## Introduction

In the last decade, photodynamic therapy (PDT) has emerged as a potential therapeutic tool for treating various tumors, and has attained elevated interest based on the noninvasive nature of the technique.^1^ The technique works *via* a combination of three components: a photosensitizer (PS) or drug, light, and oxygen. Controlled generation and deactivation of short-lived cytotoxic agents within a cell upon irradiation of a prodrug or photosensitizer is the key step in PDT.^2^ Light excitation of a dye causes an intermolecular triplet–triplet energy transfer that generates the highly reactive cytotoxic agent, singlet oxygen (^1^O_2_), within a target region, which in turn destroys the affected cells. The technique has precise spatial and temporal control and is externally switchable.^3^ However, the efficacy of the technique is limited by issues including (i) poor water solubility of photosensitizers, which leads to aggregation in aqueous media (during blood circulation) and altered photophysical, photochemical and biological properties from those otherwise expected, (ii) a low molar extinction coefficient in the far-red region of light, which is critical for deep tissue penetration, (iii) low production of singlet oxygen due to severe hypoxia caused by oxygen consumption and vascular shutdown in tumors, and (iv) non-targetability of the sensitizer that induces dark toxicity.^4^ These constraints demand novel molecular designs and delivery strategies to improve the therapeutic efficacy.^5^

Recently, targeting mitochondria, vital organelles for cell survival as they play central roles in energy production and apoptotic pathways, has been recognized as an efficient strategy in different therapeutic techniques by disturbing the normal function.^6^ Particularly in PDT, mitochondria-targeting sensitizers can overcome the hypoxia factor, resulting in high efficacy.^7^ Indocyanine dyes, mainly IR-780 derivatives, are known for their mitochondria-targeting ability and good absorption in the far-red region of light which makes them suitable for PDT applications.^8^ However, the inherent fast photobleaching, hydrophobicity, dark toxicity and low dose tolerance of the dye limit the PDT efficacy, which in turn originates from self-aggregation of the dye in aqueous media.^9^ As an alternative, a general strategy employed is encapsulation of the PS or drug in the hydrophobic core of a polymeric or lipid-based nanocarrier.^10^

**Fig. 1:**
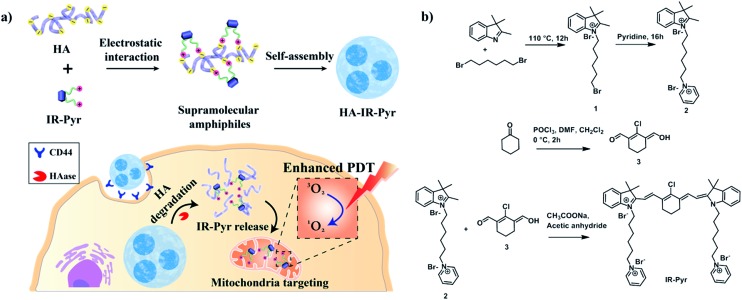
(a) Schematic representation showing the formation of **HA-IR-Pyr**, receptor mediated (CD44) cellular uptake and cancer-mitochondria localization for enhanced PDT, (b) synthetic scheme for **IR-Pyr**.

Among these, hyaluronic acid (HA), a negatively charged polysaccharide, has been extensively used for cancer selective drug delivery applications due to overexpressed HA receptors (CD44) in cancer cells.^11^ The excellent biocompatibility and unique biological characteristics of the polymer make it suitable for these applications. Herein, we have developed a water soluble indocyanine derivative, **IR-Pyr**, with preferential accumulation in mitochondria and better photostability than that of IR-780. Furthermore, electrostatic interactions between the positively charged **IR-Pyr** and the negatively charged HA polymer were used to generate micellar aggregates (**HA-IR-Pyr**) that preferentially accumulate in CD44 overexpressing tumors, are cleaved by hyaluronidase inside the cell, and localize in the cancer mitochondria (Fig. 1a) to induce high PDT efficacy during laser irradiation, which has been proven by *in vitro* and *in vivo* experiments.

## Results and discussion

### Synthesis and photophysical properties of **IR-Pyr** and **HA-IR-Pyr**

**Fig. 2:**
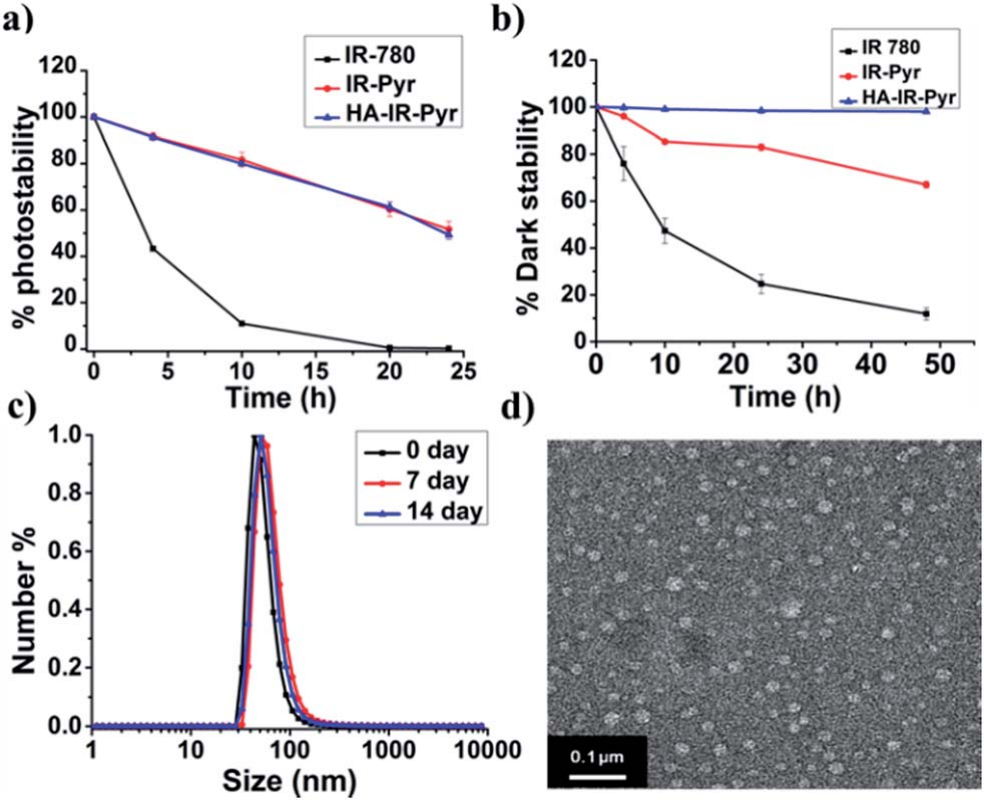
Comparison of (a) photostability and (b) dark stability in PBS. (c) DLS and (d) a TEM image for **HA-IR-Pyr**.

**IR-Pyr** was synthesized *via* a multi-step synthetic strategy (Fig. 1b). In the first step, 2,3,3-trimethylindoline was condensed with 1,6-dibromohexane to obtain compound **1**. Pyridinium ion substituted trimethylindolinium bromide (**2**) was synthesized by reacting compound **1** in excess pyridine at 110 °C. In the final step, condensation of **2** and **3** in acetic anhydride with sodium acetate gave a crude mixture of **IR-Pyr**, which was green in color. The mixture was purified by column chromatography in a silica gel column, followed by high performance liquid chromatography (HPLC) purification to yield the desired product (**IR-Pyr**). Formation of the precursors and **IR-Pyr** was confirmed using different spectroscopic techniques such as ^1^H NMR, ^13^C NMR and ESI-MS (Fig. S1–S12†). **IR-Pyr** is highly soluble in water (log *P* –0.16), which justifies the molecular design with a pyridinium ion for mitochondrial targeting. The optical behaviour of the molecule in aqueous media was investigated and showed a major absorption peak at 776 nm with a hump at 706 nm (Fig. S13†). Interestingly, the photo and dark stabilities of **IR-Pyr** were found to be significantly better in comparison with IR-780 (Fig. 2a and b), and were monitored by measuring changes in the absorption spectra with respect to time. The improved stability of **IR-Pyr** is attributed to the increased water solubility, which prevents aggregation in aqueous media.

To improve cancer selectivity, a supramolecular polymer was constructed by utilizing the electrostatic interaction between the positively charged **IR-Pyr** and the negatively charged HA polymer. The formation of micellar aggregates (**HA-IR-Pyr**) was confirmed by DLS, zeta potential and TEM analyses. The average size of the spherical micellar aggregates was 60 nm (Fig. 2c and S14†) in DLS, whereas the TEM analysis shows an average particle size of 30 nm (Fig. 2d). A negative zeta potential was observed (–40 mv), demonstrating the formation of the polymer-coated supramolecular structure (Fig. S15†). The encapsulation efficiency of the dye was calculated using absorption spectra and was found to be 0.33. Furthermore, it was found to be stable at 4 °C for up to 90 days, as determined by DLS analysis (Fig. S16†).

**Fig. 3:**
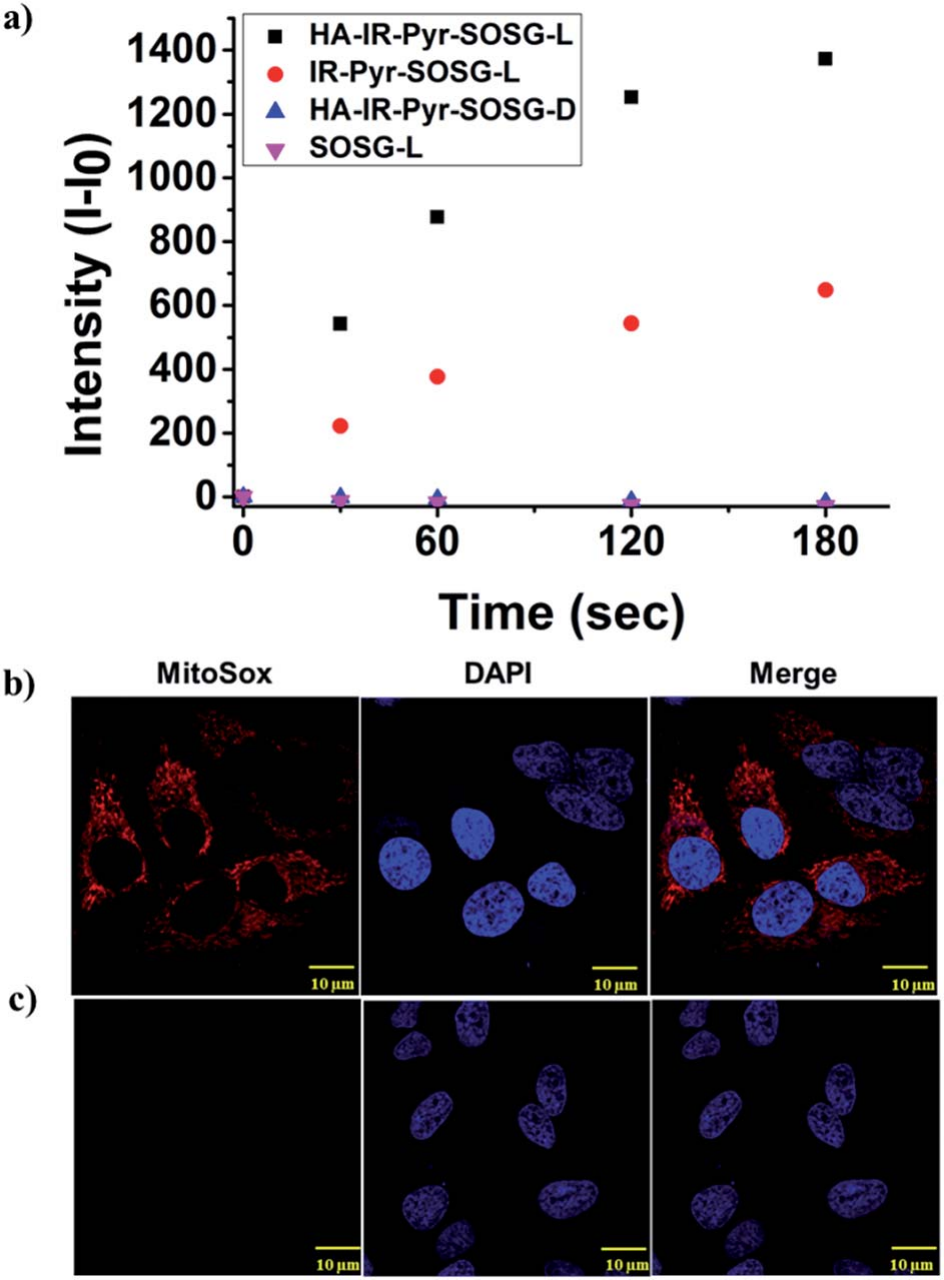
Comparison of ^1^O_2_ generation ability in (a) PBS, (b) HeLa cell lines after 3 min irradiation and (c) HeLa cell lines without irradiation.

The photostability of the micellar aggregates was similar to that of **IR-Pyr**, whereas the dark stability significantly improved upon HA coating (Fig. 2a and b). The generation of ^1^O_2_ by the micellar aggregates in PBS was investigated using an 808 nm laser with 3 min irradiation, and the results were compared with **IR-Pyr**. Singlet oxygen sensor green (SOSG) was used to monitor the singlet oxygen generation ability of the micellar aggregates, and showed an enhancement in the fluorescence intensity upon reaction with ^1^O_2_. A 10 μM solution of compounds was mixed with an equimolar solution of SOSG in independent experiments. The change in fluorescence intensity of SOSG at 530 nm was monitored after excitation at 504 nm before and after irradiating with laser light (808 nm, 200 mW cm^–2^) at different time intervals. The enhanced emission of SOSG gives direct indication of increased generation of singlet oxygen in the medium, and showed the higher ability of **HA-IR-Pyr** over **IR-Pyr** (Fig. 3a). This is possibly due to the different photophysical environment of the micellar aggregates with respect to the molecular state. The fluorescence life time of **HA-IR-Pyr** (10.55 μs) was longer than that of **IR-Pyr** (9.4 μs), and can be correlated with enhanced ROS generation in the aggregates.

### Cancer-mitochondria-targeting using **HA-IR-Pyr**

**Fig. 4:**
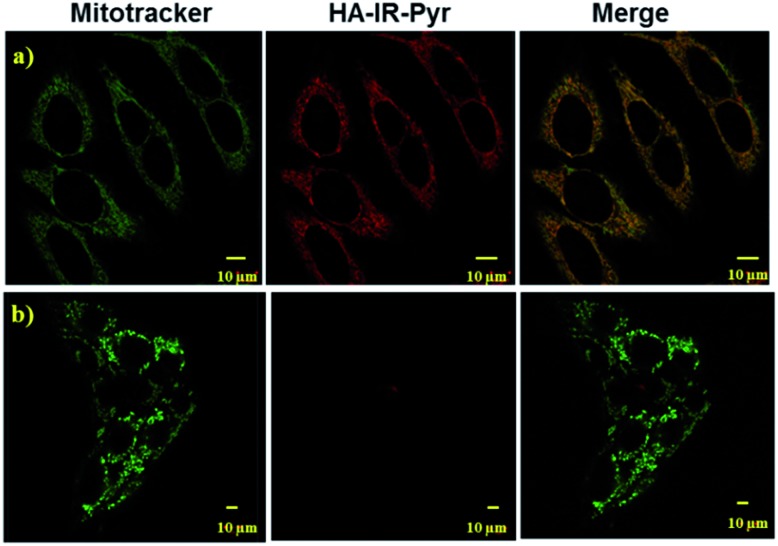
Confocal images for analyzing mitochondria co-localization of **HA-IR-Pyr** in (a) HeLa cell lines and (b) HeK-293T cell lines.

As mitochondria are important regulators of cell death, PDT induced cytotoxicity within the mitochondria is found to be a promising treatment modality. We investigated cellular uptake and mitochondrial accumulation of **IR-Pyr** in both cancerous (HeLa) and non-cancerous (HeK293T) cell lines, and compared with IR-780 (Fig. S17†). The Pearson’s co-localization coefficients for mitochondria localization were 0.61 and 0.73 for IR-780 and **IR-Pyr** respectively. However, both compounds accumulated in the non-cancerous cells as well, which is undesirable for an efficient therapeutic technique. In contrast, **HA-IR-Pyr** showed cancer selectivity and localized preferably in cancer mitochondria (Fig. 4). Confocal images after 4 h incubation with **HA-IR-Pyr** showed extensive accumulation in the HeLa cell lines (Pearson’s co-localization 0.77), while showing negligible accumulation in the HeK293T cell lines. The selectivity of **HA-IR-Pyr** toward HeLa cell lines is attributed to the overexpression of CD44 in the cell lines (Fig. S18†). Furthermore, the hyaluronidase enzyme could cleave the HA in the micellar aggregates, accelerating the release of **IR-Pyr** inside the cell.^10^

### Significance of HA and the cellular uptake mechanism

The significance of the HA coating for selective cellular uptake was further investigated with and without pretreatment of free HA. The confocal images show that cellular accumulation in the HeLa cell lines was significantly less for free HA-pretreated samples than for those that were not pretreated (Fig. S19†). However, there were no significant differences between free HA-pretreated and non-treated HeK293T cell lines, with the uptake being low in both cases (Fig. S20†). This clearly implies that the HA-coated dye selectively accumulates in cancer cell lines, which is attributed to the overexpression of HA receptors. Furthermore, the dye did not accumulate in free HA-pretreated HeLa cells, which is attributable to a competitive effect. We studied the cellular uptake mechanism in detail. Endocytosis is a common mechanism underlying the cellular uptake of nanoparticles that is implemented *via* various mediators such as clathrin, caveolae and macropinocytosis. The uptake mechanism of **HA-IR-Pyr** in HeLa cells was determined using confocal imaging with various endocytosis inhibitors such as sucrose for inhibiting clathrin mediated endocytosis, methyl-β-cyclodextrin (M-βCD) for caveolae and amiloride for macropinocytosis mediated cellular uptake. The confocal images show that the uptake mechanism is controlled by macropinocytosis and clathrin mediated endocytosis (Fig. S21†).

### *In vitro* PDT experiments

The high mitochondrial accumulation and selectivity toward cancer cells were promising enough to conduct further experiments to investigate the PDT application of the micellar aggregates. Intracellular ROS generation during photoirradiation of **HA-IR-Pyr** was confirmed using confocal imaging. **HA-IR-Pyr** (2.5 μM) was incubated with HeLa cell lines for 2 h. ROS indicator (MitoSox Red) was added before irradiation with an 808 nm laser (200 mW cm^–2^) for 3 min. The bright red fluorescence images from the confocal microscopy confirmed the generation of singlet oxygen inside the cell (Fig. 3b). A control experiment without photoirradiation did not produce any significant signal intensity indicating the photocontrolled generation of ROS (Fig. 3c). However, the control experiment with IR-780 showed no remarkable difference between the laser irradiated and non-irradiated cells (Fig. S22†), which is attributed to its inherent dark toxicity.

**Fig. 5:**
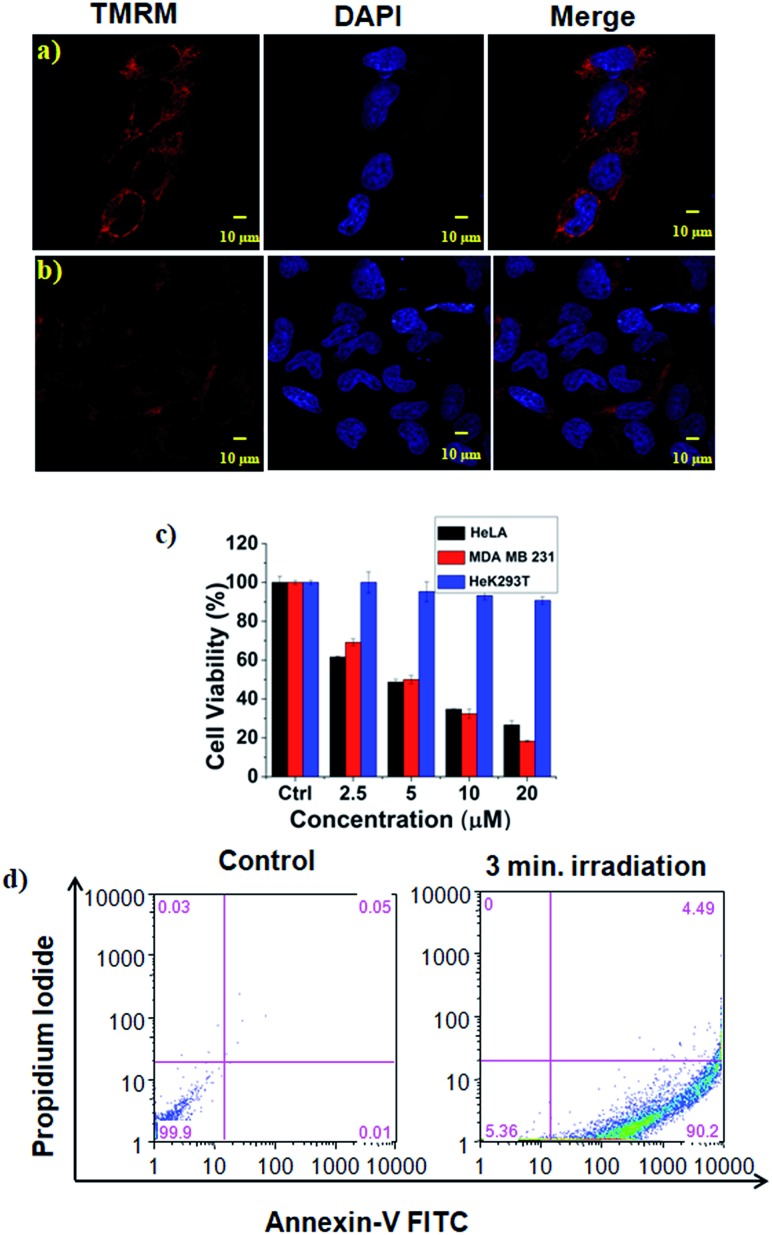
The effect of PDT on HeLa cells using **HA-IR-Pyr**. Mitochondrial membrane depolarization analysis using TMRM, (a) before laser irradiation, (b) after 3 min laser irradiation, (c) phototoxicity after 3 min laser irradiation, and (d) flow cytometry analysis comparing the extent of apoptosis before and after irradiation.

In addition, the mitochondrial damage during PDT was investigated by measuring differences in the mitochondrial membrane potential during the photoirradiation and singlet oxygen generation. Analyses of mitochondrial membrane depolarization using TMRM showed a visible difference in the HeLa cell lines during PDT with **HA-IR-Pyr**, as reflected by the fluorescence turn off from TMRM (Fig. 5b). However, without irradiation the mitochondria were found to be intact (Fig. 5a). These results confirm the PDT driven destruction of the mitochondria.

Cell viability analyses after PDT with **HA-IR-Pyr** (808 nm laser, 3 min irradiation, 200 mW cm^–2^) were conducted in HeLa, MDA-MB-231 cell lines (cancerous models), and HeK293T cell lines (non-cancerous model) (Fig. 5c and S23†). The alamar blue assay showed that **HA-IR-Pyr** induced cytotoxicity in a concentration dependent manner in the cancer lines (HeLa and MDA-MB-231) after photoirradiation, whereas it did not induce remarkable changes in the Hek293T cell lines due to a lack of accumulation in the non-cancerous model. The IC50 was found to be 5–7 μM in both the HeLa and MDA-MB-231 cell lines, whereas dark control experiments showed more than 85% viability, implying the efficient PDT effect of **HA-IR-Pyr**. In contrast, IR-780 showed significant dark toxicity in the HeLa, MDA-MB-231 and HeK293T cell lines (Fig. S24†). Furthermore, the extent of apoptosis during PDT was determined by flow cytometry analysis using propidium iodide and annexin V. These data showed 90% apoptosis population (Fig. 5d) after PDT with **HA-IR-Pyr**, whereas the dark control experiment showed no significant apoptotic cell population, inferring apoptosis mediated cell death during PDT.

### *In vivo* imaging and PDT experiments

**Fig. 6:**
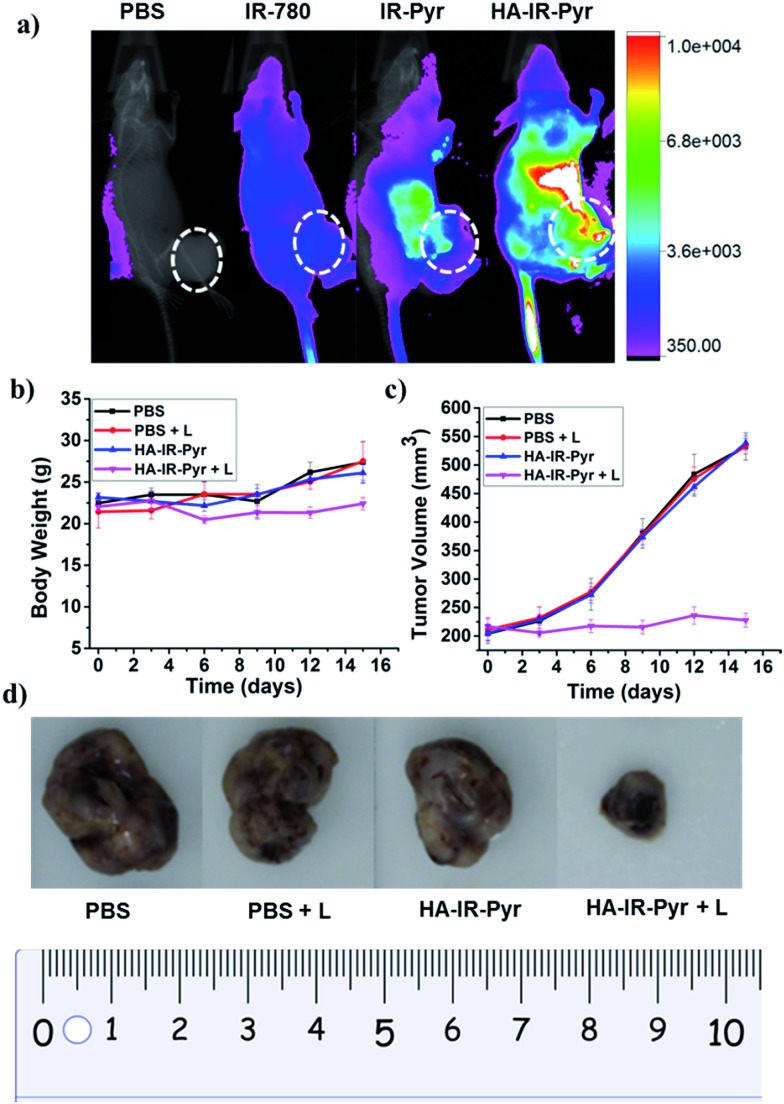
(a) Accumulation of **HA-IR-Pyr** in an SSC7 tumor model after 8 h in comparison with other control molecules, (b) changes in body weight with time during PDT, (c) change in the tumor volumes with time during PDT and (d) comparison of the tumor size after 16 days.

*In vivo* tumor imaging with **HA-IR-Pyr** was performed in a SCC7 tumor xenograft model, which is important in PDT to localize the tumor and external time control on irradiation. The tumor imaging ability was compared with **IR-Pyr** and IR-780. The compounds were intravenously injected into tumor-bearing mice (one mouse per group) and imaged at several time points using an optical imaging system by setting the excitation at 760 nm and the emission at 830 nm. We found that **HA-IR-Pyr** preferentially accumulated in the tumor after 6 to 8 h, whereas the control molecules (**IR-Pyr** and IR-780) did not accumulate in the tumors to show sufficient fluorescence signals (Fig. 6a and S25†). The increased accumulation of **HA-IR-Pyr** was attributed to the overexpressed CD44 in the SCC7 tumor model, which is in agreement with the *in vitro* studies using CD44 positive cells.

The PDT efficacy of **HA-IR-Pyr** was investigated *in vivo* after injecting the micellar aggregates intravenously into SCC7 tumor-bearing mice. An NIR laser (808 nm) was selected and low power irradiation (200 mW cm^–2^) was used as an excitation source to activate **HA-IR-Pyr** for *in vivo* PDT. Four groups of tumor-bearing mice were subjected to different treatments: PBS + L (with light irradiation), PBS (without light irradiation), **HA-IR-Pyr** + L (with light irradiation) and **HA-IR-Pyr** (without light irradiation) with four mice per group. **HA-IR-Pyr** accumulation at the tumor sites reached its maximum at 8 h, at which time the selected groups were irradiated with an NIR laser (200 mW cm^–2^) for 3 min. Phototoxicity of the micellar aggregates to the tumors was assessed by monitoring the relative tumor volumes and body weight changes. As shown in Fig. 6b, no significant changes in body weight were noted in the **HA-IR-Pyr** and control groups with and without laser irradiation, indicating minimum side effects during PDT with the micellar aggregates.

**Fig. 7:**
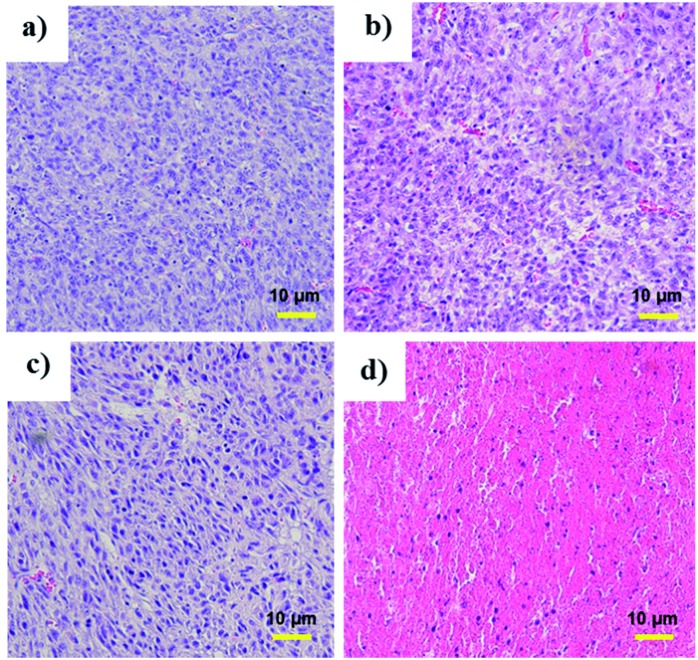
H and E stained sections of tumors after treatment with, (a) PBS (b) PBS + L, (c) **HA-IR-Pyr** and (d) **HA-IR-Pyr** + L.

Tumor volume measurements showed clear differences in tumor growth between the **HA-IR-Pyr** + L group and the control groups (Fig. 6c and d, S26 and S27†). No tumor reduction was observed in the control groups (PBS + L and PBS). Similarly, **HA-IR-Pyr** (dark control) showed negligible toxicity in the dark. In marked contrast, significant tumor growth inhibition was observed in the **HA-IR-Pyr** + L group and the tumor tissue showed obvious cell death. The treatment efficiency, in terms of tumor cell death was also evaluated by hematoxylin and eosin (H & E) staining of tumor tissue sections. After 16 d, both the PDT treated and the untreated mice were sacrificed for H & E staining. Noticeable signs of difference were observed between the control groups [PBS + L, PBS and **HA-IR-Pyr**] and the **HA-IR-Pyr** + L group, as shown in Fig. 7. These results confirm the high PDT efficacy of the **HA-IR-Pyr** micellar aggregates in the *in vivo* model with minimum dark toxicity.

## Conclusions

In conclusion, we developed **IR-Pyr**, a mitochondria targeting, water soluble indocyanine dye for PDT application. Incorporation of a pyridinium ion into the indocyanine skeleton increased the water solubility which prevents aggregation and provides increased photo and dark stabilities over those of IR-780. The overall positive charge was increased from one to three which enhanced the mitochondria targeting ability in comparison with IR-780. Furthermore, the construction of HA coated micellar aggregates **HA-IR-Pyr** provided cancer targeting ability that lead to cancer-mitochondria-targeted PDT. The singlet oxygen generation efficiency of the micellar aggregates was better than that of the free dye **IR-Pyr**, reflecting results of the *in vitro* and *in vivo* PDT experiments. These results discussed here showcase the importance of mitochondria targeted PDT, and would aid in the development of molecular systems with higher therapeutic efficacy.

## Conflicts of interest

Authors declare no competing financial interest.

## Supplementary Material

Supplementary information
