# Supplementary material for: Cancer-mitochondria-targeted photodynamic therapy with supramolecular assembly of HA and a water soluble NIR cyanine dye
Source: Chem Sci. 2017 Oct 13;8(12):8351–6. doi: 10.1039/c7sc03169f (PMC5858757; doi:10.1039/c7sc03169f)
Supplement: Supplementary file 1 [file SC-008-C7SC03169F-s001.pdf]

Electronic Supplementary Material (ESI) for Chemical Science.
This journal is © The Royal Society of Chemistry 2017

Supporting Information

Cancer-mitochondria-targeted photodynamic therapy with supramolecular assembly of HA and a water soluble NIR cyanine dye

Ajesh P. Thomas,[†] L. Palanikumar,[†] M.T. Jeena, Kibeom Kim, and Ja-Hyoung Ryu*

[†] These authors contributed equally to this work

Materials and methods

General Information:

The reagents and materials for the synthesis were used as obtained from Sigma - Aldrich and Alfa Aesar chemical suppliers. Sodium hyaluronate was purchased from Acros Organic (Belgium). All solvents were used after drying by standard methods prior to use. The NMR solvents were used as received and the spectra were recorded with Agilent 400 MHz spectrometer. Spectra were referenced internally by using the residual solvent (^1H δ =3.34 and ^{13}C δ = 49.86 for CD_3OD-d4) resonances relative to SiMe4. The ESI-MS spectra were recorded on Bruker, 1200 Series & HCT Basic System. The electronic absorption spectra and steady state fluorescence spectra were recorded on Jasco V-670 spectrophotometer and Hitachi F-7000 fluorescence spectrophotometer respectively. DLS was measured in a Malvern-Zetasizer nano system.

Cell culture and cell viability:

Human cervical cancer HeLa cells and non-cancerous fibroblast HeK293T cells were cultured (using DMEM medium) supplemented with 10% fetal bovine serum (FBS), 100 μgmL^{-1} streptomycin and 100 U mL^{-1} penicillin in sterile 96-well Nunc (Thermo Fisher Scientific Inc.) microtitre plate at a seeding density of 5 x 10^3 cells/well and they were allowed to settle for 24 h under incubation at 37 °C and 5% CO_2. In-order to check cell viability, the cells was then treated with different concentrations of Ir-780, **IR-Pyr** and **HA-IR-Pyr** (2.5, 5.0, 10.0 and 20 μM) for 12 h incubation, washed, replaced with fresh media and checked the cell viability after another 12 h incubation using the alamar blue dye assay by setting the excitation wavelength at 565 nm and monitoring emission at 590 nm excitation under dark and in presence of laser irradiation after 3 min at 200 $mWcm^{-2}$.

Cellular uptake analysis:

HeLa and HeK293T cells were seeded in one well glass cover glass (Lab Tek II, Thermo Scientific) at a seeding density of 2 x 10^5 cells/well. After 24 h, cells were treated with 2.5 μM for a period of 4 h and replaced with fresh media. The cellular uptake was monitored periodically using Carl Zeiss LSM 780 NLO multiphoton microscope connected to CO_2 incubator setting the excitation at 720 nm and emission between 725-758 nm along with the colocalization analysis with mitotracker green FM setting excitation at 488 nm and emission between 500-550 nm.

Endocytic pathway analysis.

To check the endocytosis mediated uptake, HeLa cells were seeded in chambered cover glass and pretreated with different endocytosis inhibitors including sucrose (clathrin-mediated uptake, 400 nM), methyl-β cytodextrin (caveolae mediated uptake) and amilorin (macropinocytosis) in serum-free DMEM for 1 h and replaced with fresh media. Afterwards, the uptake pathway for **HA-IR-Pyr** at 2.5 μM for a period of 4h and analyzed using the Carl Zeiss LSM 780 NLO Multiphoton microscope connected to CO_2 incubator setting the excitation at 720 nm and emission between 725 nm to 758 nm.

MitoSox ROS generation analysis.

HeLa and HeK293T cells were seeded on a Lab Tek II chamber cover glass at 90% confluence in DMEM media supplemented with 10% FBS, 100 μg mL^{-1} streptomycin, 100 UmL^{-1} penicillin and incubated at 37 °C under 5% CO2. After incubation with 2.5 μM of HA-IR-Pyr for different time intervals, by following the manufacturer's protocol (MitoSox, M36008); the cell culture medium was then replaced with media containing 5 μM MitoSox reagent working solution to cover the adherent cells. The cells were then incubated for 10 minutes at 37 °C, protected from light and irradiated using 200 $mWcm^{-2}$. The cells were then analyzed under a FV1000 laser confocal scanning microscope.

TMRM depolarization analysis:

Confocal imaging of TMRM depolarization in HeK293T and HeLa cells were pre-incubated 200 nM of TMRM for 30 min and incubated with 2.5 μM of **HA-IR-Pyr** and analyzed using an FV1000 laser confocal scanning microscope at different time intervals.

In vivo imaging with tumor xenografts:

Nude mice bearing SCC7 cancer (tumor volume ~ 200 mm^3) were intravenously injected with PBS (100 μL), IR-780 (10 μg/100 μL), IR-Pyr (10 μg/100 μL) and HA-IR-Pyr (10 μg/100 μL) with each mice per group. All animal experiments were conducted under protocols approved by the Institutional Animal care and Use Committee of Ulsan National Institute of Science and Technology (UNIST). Bruker in-vivo Xtreme optical imaging system equipped with, warm air delivery to regulate animal temperature; light-tight ports for catheter injections and compatible with gas anesthesia systems was used for imaging, under standard anesthesia (Isoflurane) condition. Then the images were captured using the in vivo optical imaging system (Bruker Xtreme model) by setting the excitation at 760 nm and 830 nm as emission, with standard X-ray background at periodic intervals.

Cancer xenograft model establishment and phototherapy:

Balb/c nude female mice were purchased from the Orient bio, Korea and all animal experiments were conducted under protocols approved by the Institutional Animal care and Use Committee of Ulsan National Institute of Science and Technology (UNIST). The mouse squamous cell carcinoma (SCC7) xenograft model was induced by subcutaneously injecting SCC7 cells (0.1 mL, $1 * 10^{7}$ cells/ 100 μL in 1x PBS) into the right flank area of each nude mouse. Periodically tumor growth was monitored using the digital caliper. Upon reaching the tumor size to 200 mm^3, calculated by the formula volume = (tumor length) x (tumor width)^2/2, the mice with larger or smaller size were not selected. Four representative mice groups were selected with four mice per group and received intravenously injection mode for

PBS, PBS + L, HA-IR-Pyr and HA-IR-Pyr + L. The mice treated were with equal volume of PBS (100 μL) and HA-IR-Pyr (10 μg/100 μL). For PDT treatments, mice (+ laser) groups was irradiated using a NIR laser (808 nm, 200 $mWcm^{-2}$) for 3 min, after 8 h of I.V. injection.

Synthetic procedure and spectral characterization:

Synthesis of 1: 2,3,3-trimethyl indoline (2 g, 12.56 mmol) was mixed with excess 1,6-dibromo hexane (15.32 g, 62.8 mmol) in a 50 ml round bottom flask with a magnetic stirrer bar. The mixture was heated at 110 °C for 12 h. Solution was allowed to come to room temperature. The precipitate formed is collected and washed with diethyl ether and then hexane for several times to remove unreacted starting materials. Dried in vacuum for overnight to afford **1** in 85% yield. ^{1}H NMR (400 MHz, CD_3OD, 298K): δ = 7.70 (d, 2H), 7.49 – 7.53 (m, 4H), 4.69 (t, 2H), 2.76 (t, 2H), 1.90-1.93 (m, 2H), 1.78-1.81 (m, 2H), 1.58 (s, 6H), 1.47-1.55 (m, 4H); ^{13}C NMR (100 MHz, CD_3OD): δ = 196.08, 141.6, 140.94, 130.65, 129.49, 123. 27, 117.53, 115.44, 54.61, 49.41, 33.76, 33.68, 32.46, 32.21, 27.98, 27.64, 27.24, 25.86, 23.12, 22. 63, 16.43. ESI-MS: m/z calculated for $C_{17}H_{25}BrN^{+}$ = 322.12; found = 323.65.

Synthesis of 2: 10 ml pyridine was added to **1** (1 g, 2.48 mmol) in a RB flask, and the solution was heated overnight at 90 °C. Excess solvent was removed in roto evaporator, washed three times with hexane and ethyl acetate. Dried in high vacuum afforded **2** in 90% yield. ^{1}H NMR (400 MHz, CD_3OD, 298K): δ = 9.22-9.21 (d, 4H), 8.68-8.66 (m, 2H), 8.21-8.17 (m, 4H), 7.67-7.65 (dd, 1H), 4.82-4.77 (m, 4H), 4.72 (s, 2H), 4.63-4.59 (t, 1H), 2.14-2.10 (m, 5H), 1.64 (s, 6H), 1.55 (s, 3H); ^{13}C NMR (100 MHz, $CDCl_3$): δ = 196.46, 145.47, 144.54, 141.96, 141.65, 141.02, 129.79, 129.09, 128.11, 127.28, 123.22, 115.05, 61.44, 54.52, 30.72, 27.22, 25.64, 25.28, 21.32. ESI-MS: m/z calculated for $C_{22}H_{30}N_{22}^{+}$ = 322.49; found = 322.06).

Synthesis of 3: Dry dimethylformamide (DMF) and dichloromethane (CH_2Cl_2) was mixed together in 1:1 ration (10 ml each). The mixture was cooled in an ice bath to 0 ^oC. Phosphorous oxychloride (9 ml) was dissolved in 10 ml CH_2Cl_2 and added slowly to the mixture at 0 ^oC with constant stirring. After 15 min. cyclohexanone (2.5 g, 25.4 mmol) in 6 ml CH_2Cl_2 was added. Ice bath was removed to bring the solution to room temperature. Then refluxed for 3h, and then poured to a beaker containing 50g of ice and allowed to come to room temperature. The lower layer was drawn off using a separatory funnel, compound in the aqueous layer was filtered using a vacuum pump. The yellow precipitate was washed many times with cold water to get the desired compound in 40% yield. ^1H NMR (400 MHz, $CDCl_3$, 298K): δ = 9.94 (s, 1H), 9.74 (d, 1H), 7.3 (s, 1H), 2.24(d, 2H), 2.13(t, 2H), 1.43-1.40(m, 2H); ^{13}C NMR (100 MHz, $CDCl_3$): δ = 190.62, 152.65, 145.63, 129.56, 114.59, 24.19, 23.93, 20.22. ESI-MS: m/z calculated for $C_{18}H_9ClO_2$ = 172.6; found = 171.82.

Synthesis of IR-Pyr: A solution of **2** (0.15 g, 0.87 mmol), **3** (0.924 mg, 1.66 mmol) and sodium acetate (0.157 mg, 1.66 mmol) was dissolved in acetic anhydride (18 ml) and heated at 70 ^oC for 1h. The green color solution formed was cooled to room temperature, and poured into a saturated solution of NaBr (20 ml). Solvent was removed. The green color solid obtained was dissolved in methanol and a silica gel column was performed followed by purification by HPLC (methanol: H_2O) to get the pure compound in 45% yield. ^1H NMR (400 MHz, CD_3OD, 298K): δ = 9.00(t, 4H), 8.60-8.57(m, 2H), 8.46-8.42 (dd, 2H), 8.10 (t, 4H), 7.53-7.51 (dd, 2H), 7.43-7.40 (dd, 2H), 7.34-7.28 (m, 4H), 6.3-6.26 (d, 2H), 4.66-4.62 (m, 4H), 4.19(t, 4H), 2.72 (t, 4H), 2.06-2.04 (m, 4H), 1.95 (t, 2H), 1.85-1.89 (m, 4H), 1.71 (d, 12H), 1.56-1.47 (m, 8H); ^{13}C NMR (100 MHz, CD_3OD): δ = 172.80, 160.23, 159.86, 145.45, 144.51, 142.19, 141.18, 128.62, 128.48, 128.39, 128.09, 126.55, 125.17, 122.13, 117.50, 110.87, 100.92, 61.54, 49.22, 43.35, 30.87, 26.91, 26.88, 26.08, 25.94, 25.59, 22.8, 20.71,

19.9; ESI-MS: m/z calculated for $C_{52}H_{64}ClN_4^{3+}$ = 259.83; found = 260.00. 446.4 $(C_{29}H_{35}ClN_2)^+$.

Composition and CMC of HA-IR-Py

To prepare HA-IR-Pyr, HA (10 mg) was dissolved in 15 ml of DI water. To this solution, IR-Pyr (5 mg, dissolved in 1 ml DI water) was added slowly with continuous stirring. Further, diluted by adding another 5 ml DI water and allowed to stir for overnight at room temperature. The solution was centrifuged to remove any precipitated particles and dialyzed using a 3.5KD cut off membrane tube in DI water for 24h. The composition of micelles was 1:2 weight ratio of IR-Pyr and HA.

NMR spectral analysis:

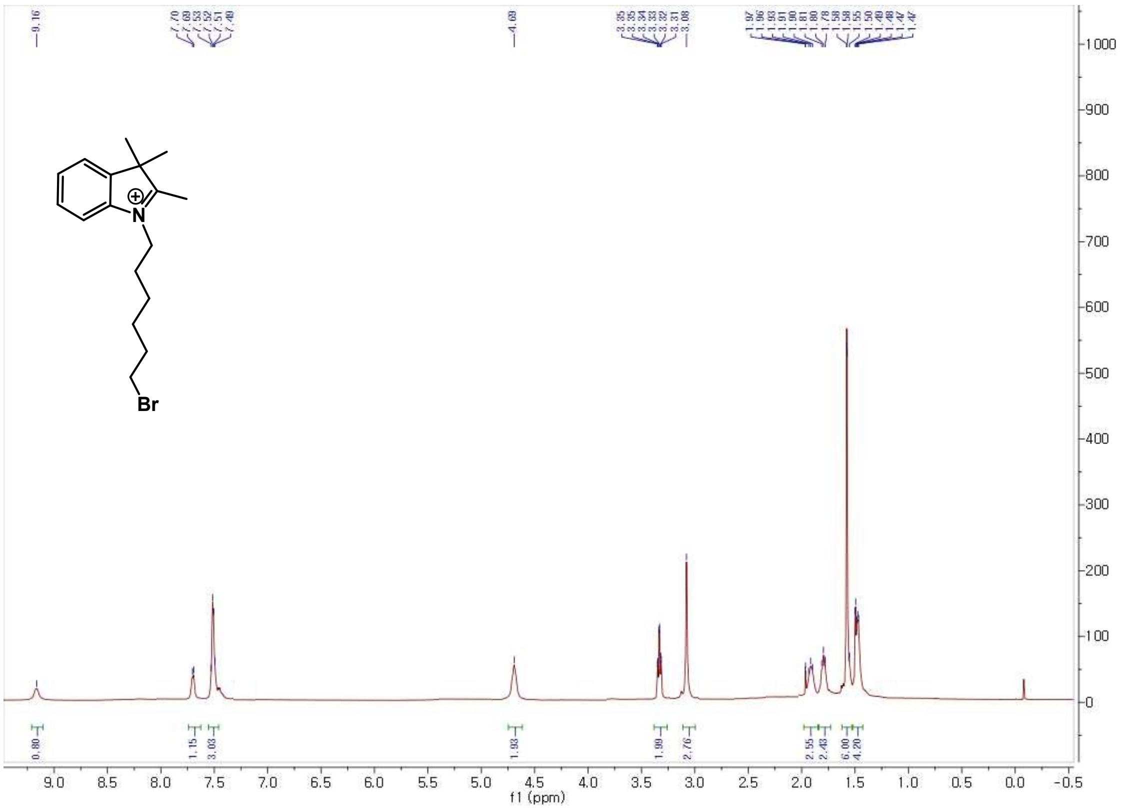

Fig. S1 ^{1}H-NMR spectrum of **1** in CD_3OD.

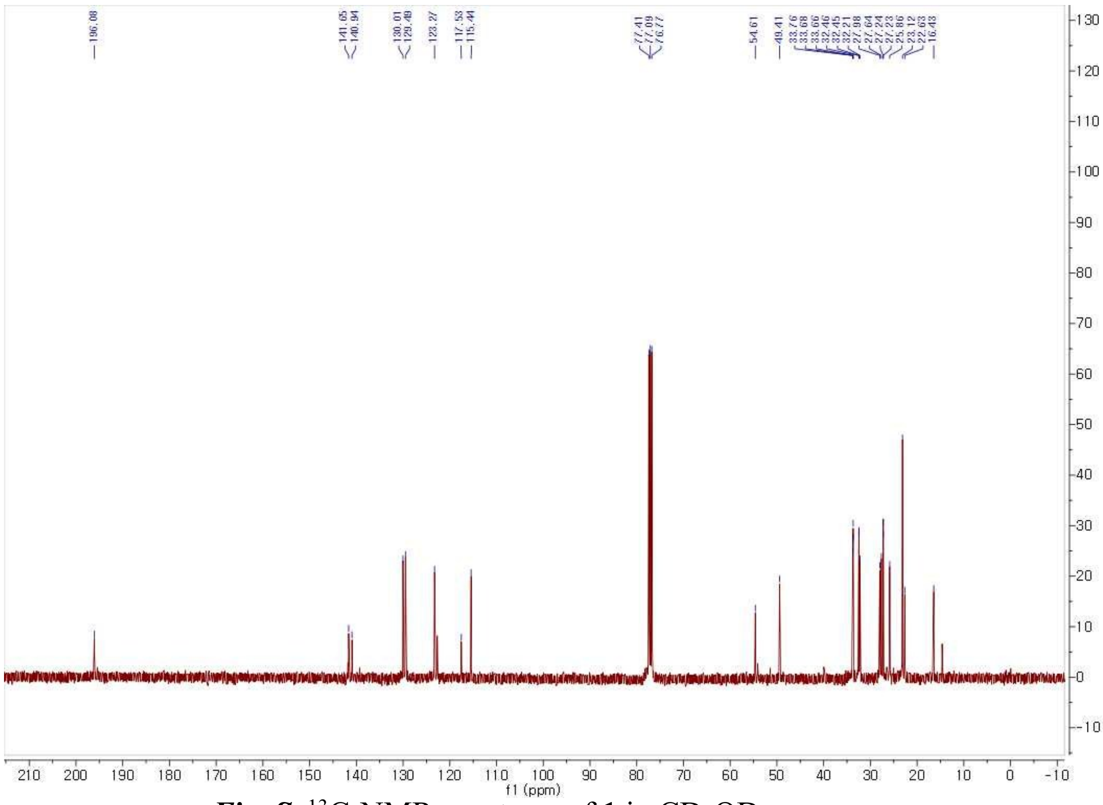

Fig. S. ^{13}C-NMR spectrum of **1** in CD_3OD.

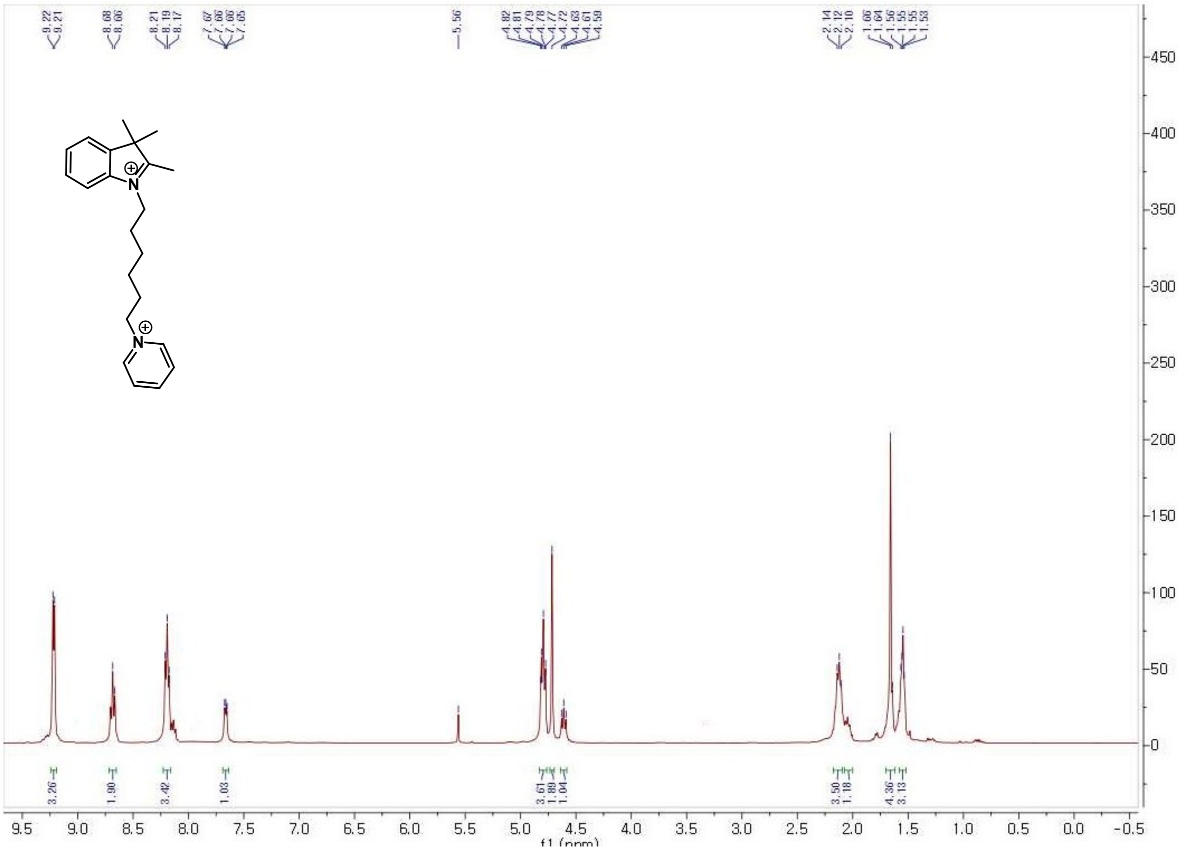

Fig. S3 ^{1}H-NMR spectrum of **2** in CD_3OD.

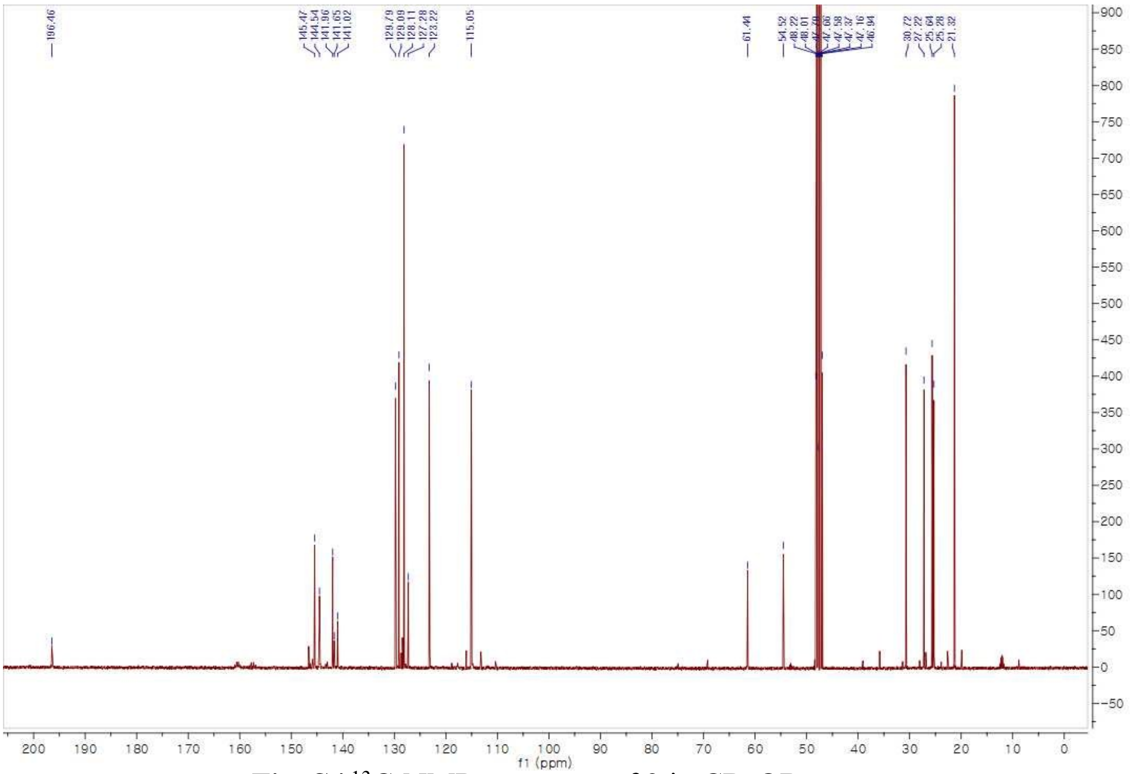

Fig. S4 ^{13}C-NMR spectrum of **2** in CD_3OD.

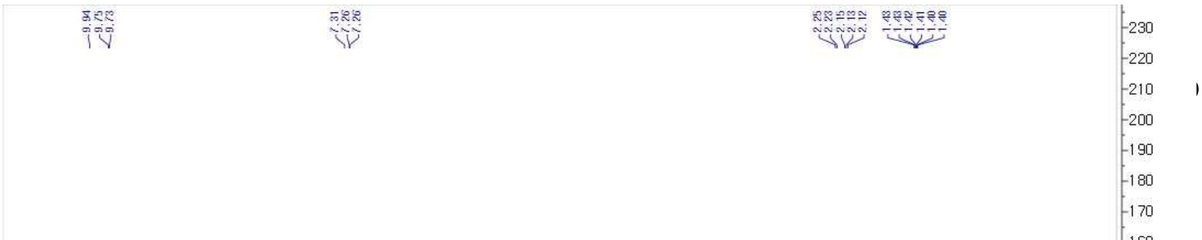

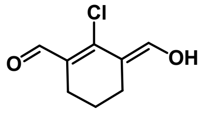

Fig. S5 ^{1}H-NMR spectrum of **3** in $CDCl_3$.

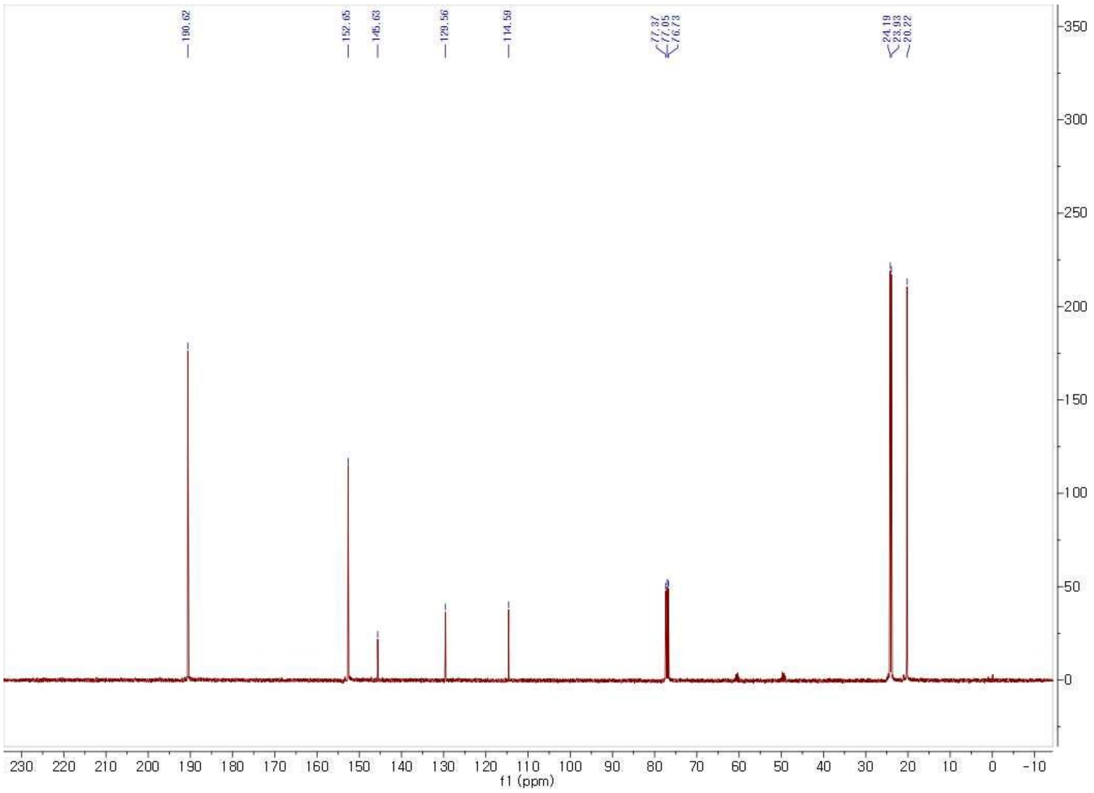

Fig. S6 ^{13}C-NMR spectrum of **3** in $CDCl_3$.

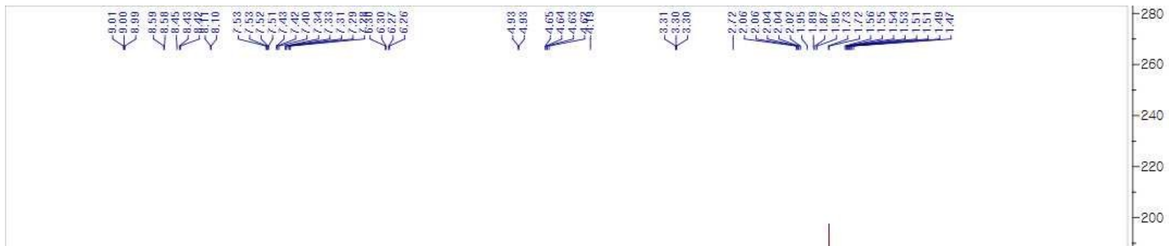

)

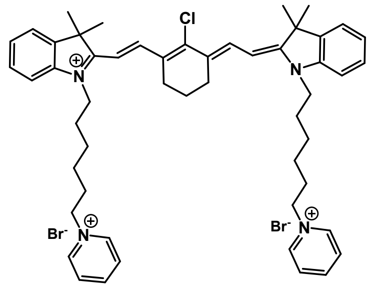

Fig. S7 ^{1}H-NMR spectrum of **IR-Pyr** in CD_3OD.

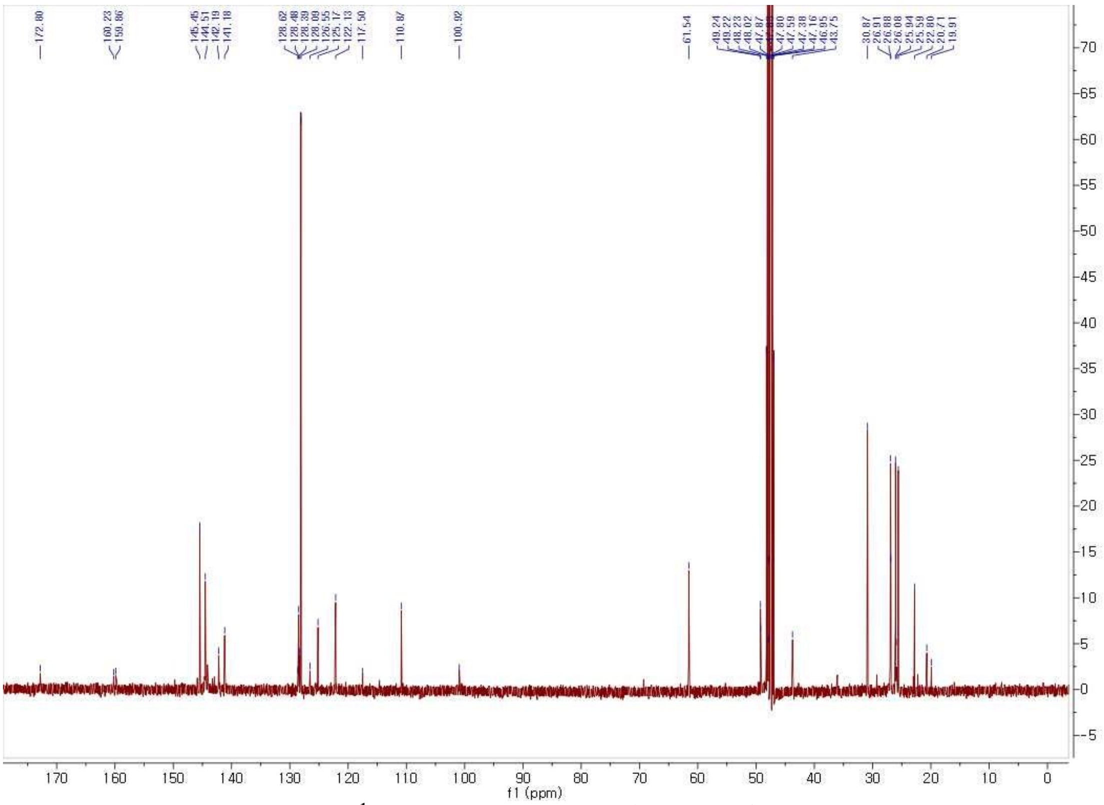

Fig. S8 ^{1}H-NMR spectrum of **IR-Pyr** in CD_3OD.

4. ESI-MS analysis

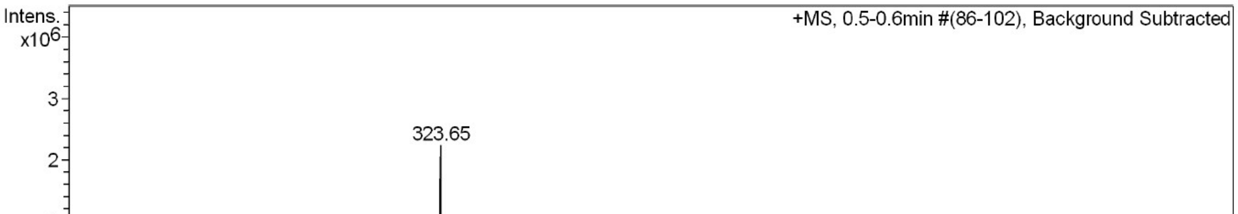

Fig. ***S9*** ESI-MS spectrum of **1.**

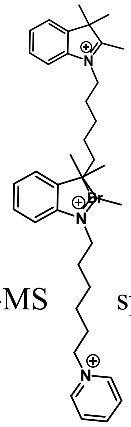

Chemical Formula: $C_{17}H_{25}BrN^{+}$
Exact Mass: 322.12

Chemical Formula: $C_{22}H_{30}N_2^{2+}$
Exact Mass: 322.24

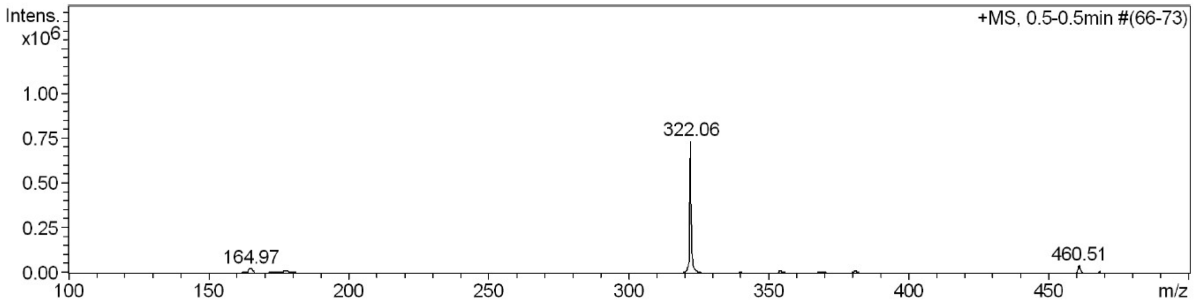

#	m/z	I
1	163.45	10623
2	164.97	28814
3	177.71	16833
4	179.81	9823
5	322.06	735584
6	339.70	9435
7	354.07	14342
8	380.87	13957
9	460.51	44185
10	467.95	12139

Fig. S10 ESI-MS spectrum of **2.**

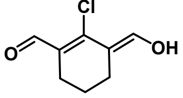

Chemical Formula: C8H9ClO2
Exact Mass: 172.03

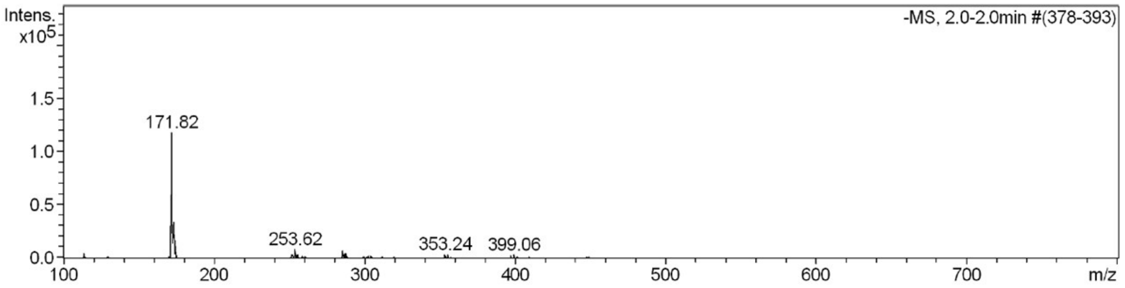

#	m/z	I
1	113.71	3890
2	171.82	117808
3	173.35	33559
4	253.62	8118
5	255.46	3267
6	285.54	7271
7	287.31	4286
8	353.24	3435
9	355.17	3345
10	399.06	3175

Fig. S11 ESI-MS spectrum of **3.**

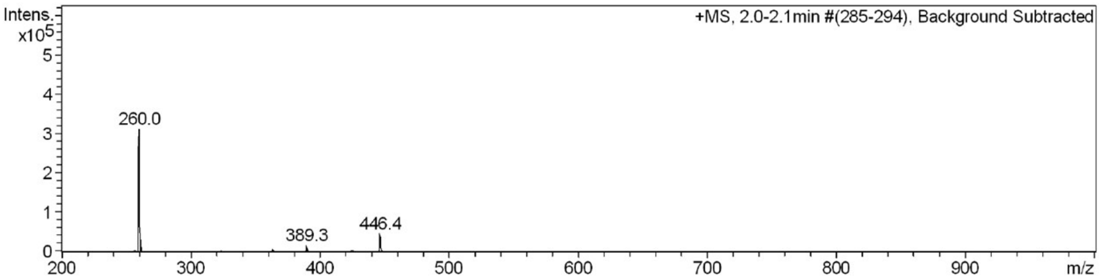

#	m/z	I
1	251.0	1544
2	256.2	2604
3	260.0	311327
4	321.2	1033
5	323.2	2980
6	363.3	7304
7	364.1	2304
8	389.3	15811
9	424.7	3504
10	446.4	46194

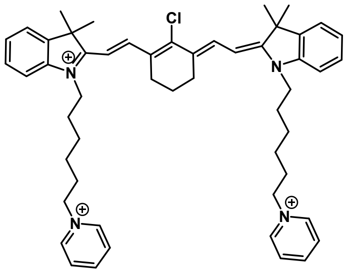

Chemical Formula: $C_{52}H_{64}ClN_4^{3+}$
Exact Mass: 779.48

Fig. S12 ESI-MS spectrum of **IR-Pyr**.

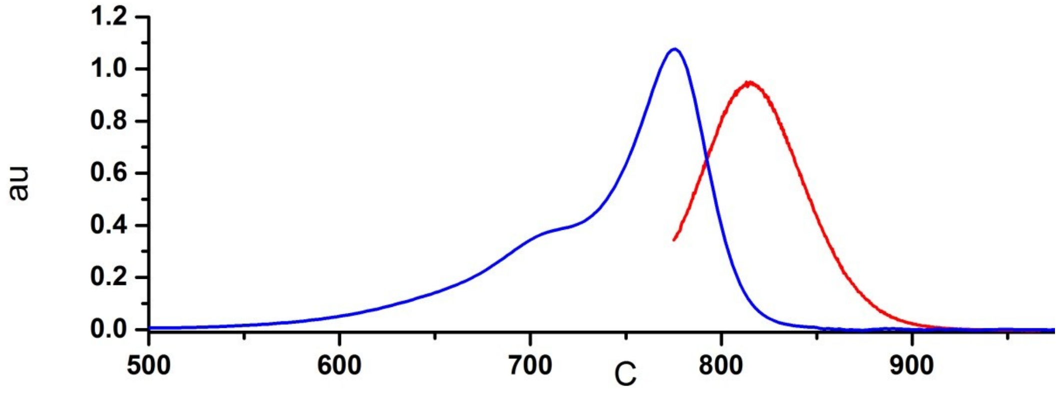

Fig. S13 Absorption (blue) and emission spectra (red) of IR-Pyr in PBS.

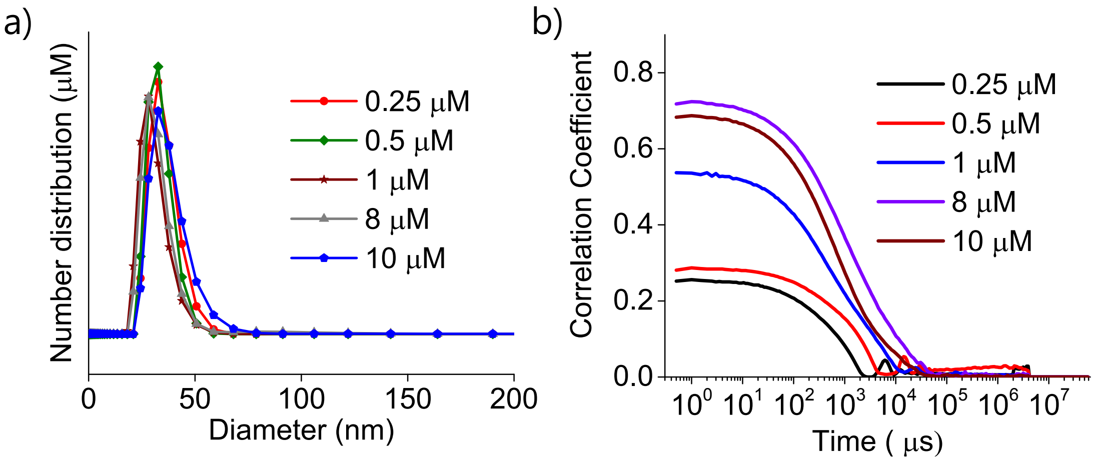

Fig. S14 a) Size distribution analysis of HA-IR-Pyr at different concentration showing similar size. b) Correlation coefficient at different concentration. To measure the CMC, we have tried several methods to evaluate the CMC of HA-IR-Pyr. We tried the well-known dye encapsulation methods (Nile red or Pyrene encapsulation), but failed to obtain meaningful data and CMC value. We have performed concentration dependent size distribution analysis. Interestingly, we found that there was no difference in the size distribution in accordance with the concentration of the micelle (Figure S14a). Further, we observe that the correlation functions have not disappeared even below 0.25 μM of IR-Pyr concentration in the micelle (Figure S14b). These observations suggest that once the micelle formed by the electrostatic interaction (between the positively charged IR-Pyr and negatively charged HA) and h[illegible] stability and the C[illegible]

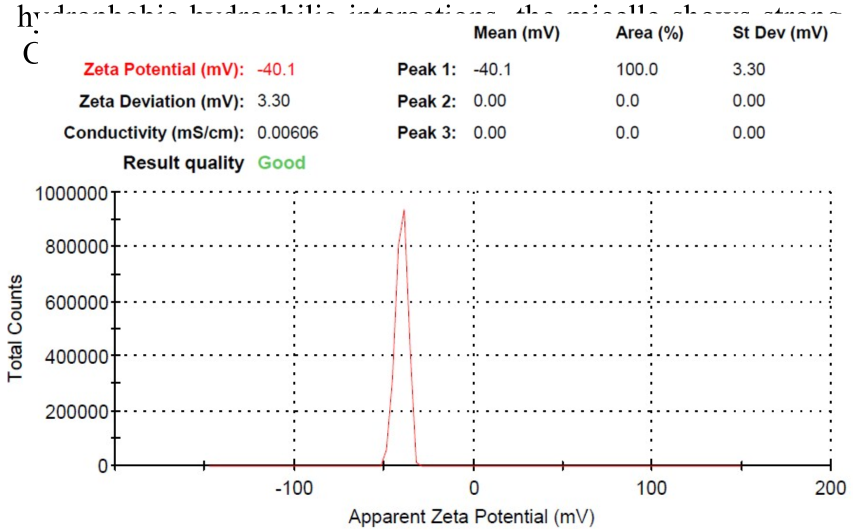

Fig. S15 Zeta potential of **HA-IR-Pyr**

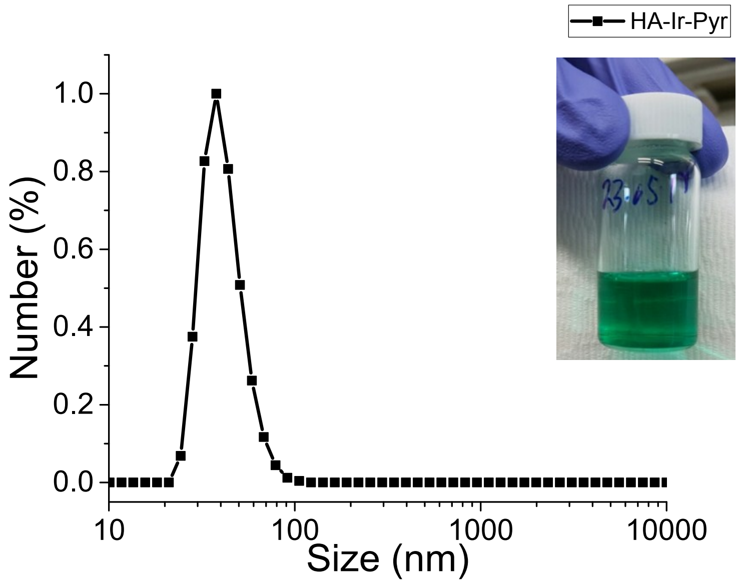

Fig. S16 Stability analysis in terms of size after 90 days of storage. Inset pictures of HA-Ir-Pyr micelle after 90 days.

In-vitro experiments

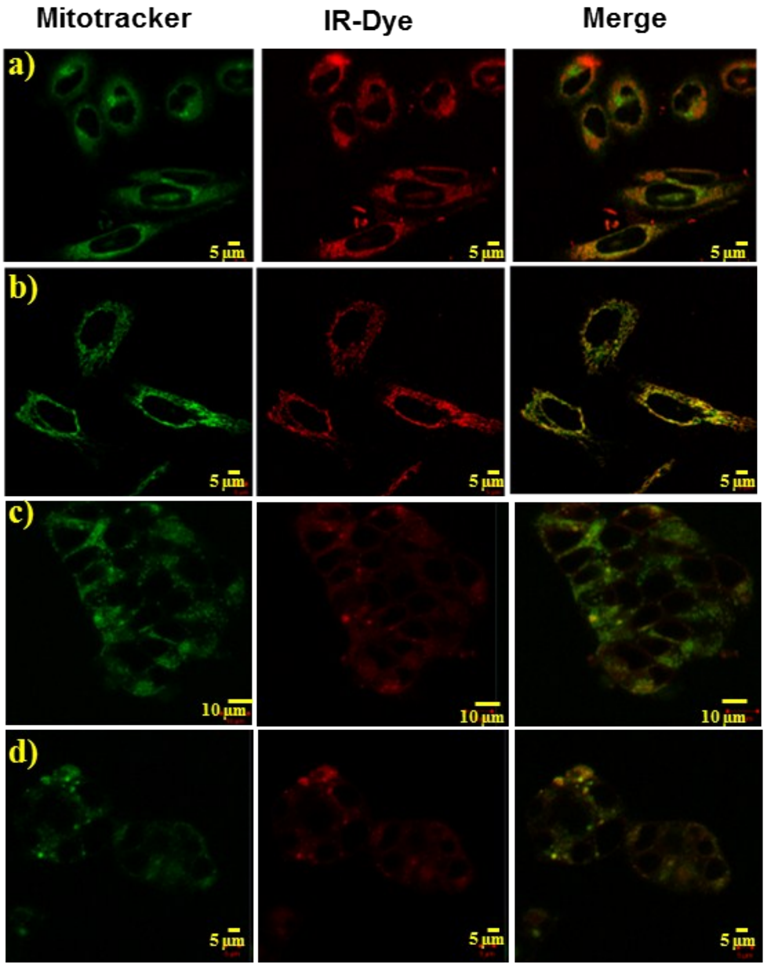

Fig. S17 Confocal images for analyzing mitochondria colocalization, a) IR-780, b) IR-Pyr in HeLa cell lines and c) IR-780 and d) IR-Pyr in HeK293T cell lines.

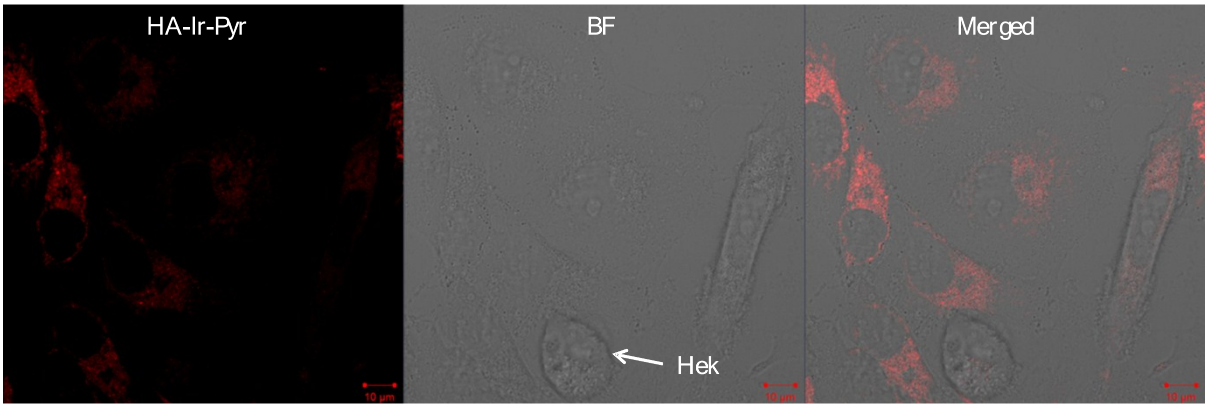

Fig. S18. Selective targeting capability of HA-IR-Pyr (2.5 μM) in HeLA and HeK293T cells after 4h incubation. Confocal microscope images to show the targeting capability analysis of HA-Ir-Pyr in CD-44 over-expressed HeLa cells and non-targeted HeK293T cells

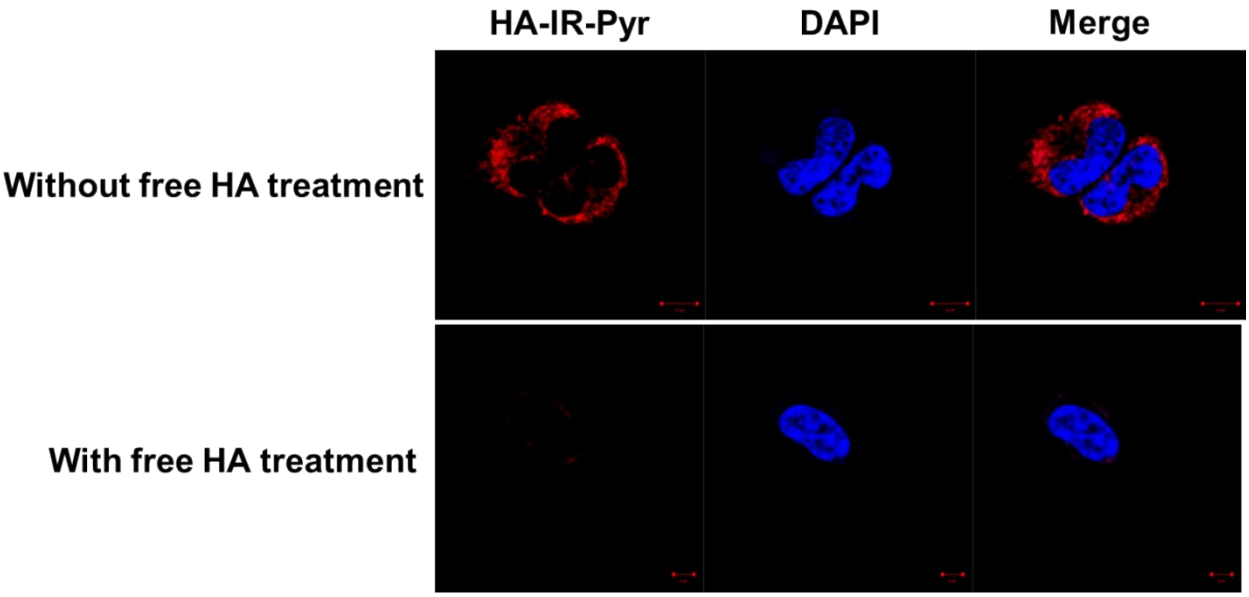

Fig. S19 Confocal images in HeLa cell lines, showing role of HA in cellular uptake.

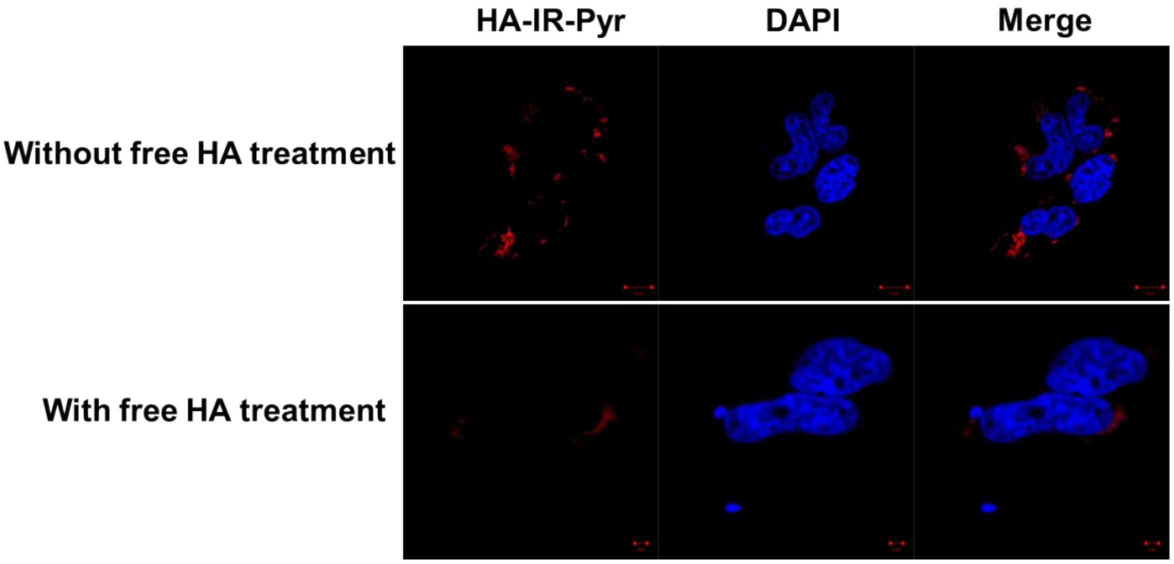

Fig. S20 Confocal images in HeK-293T cell lines, showing role of HA in cellular uptake.

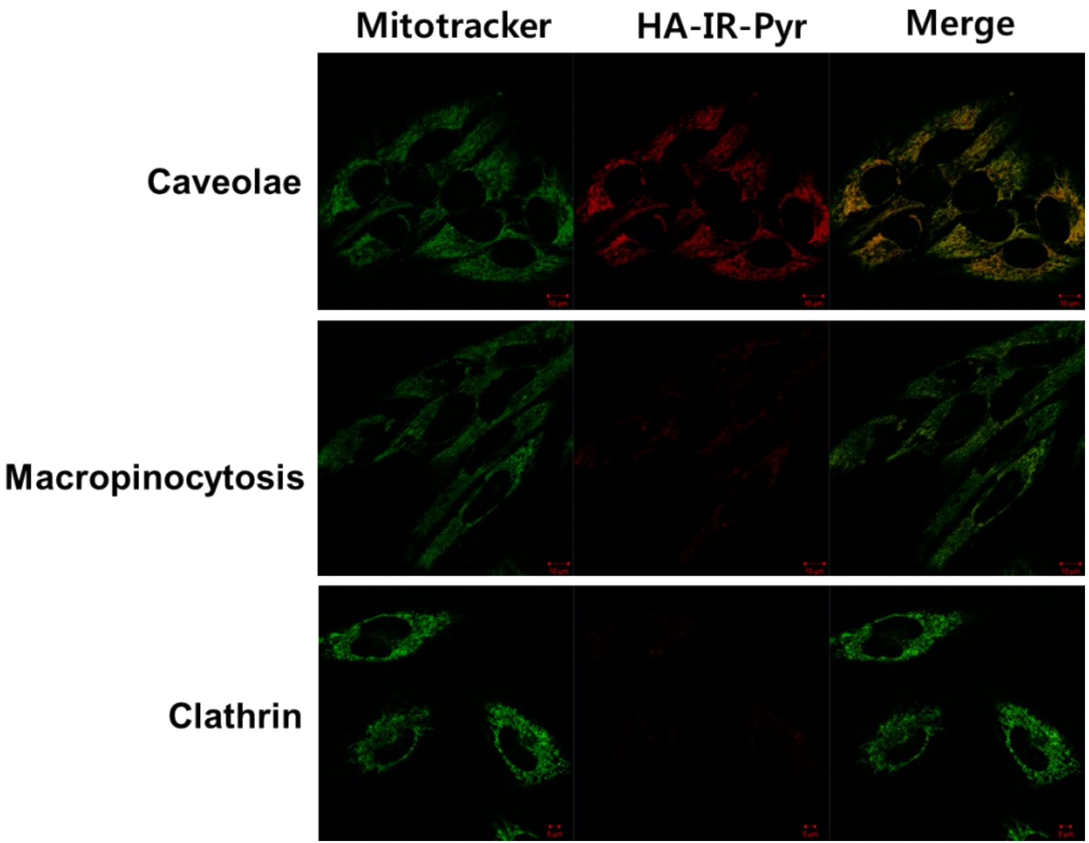

Fig. S21 Confocal images with **HA-IR-Pyr** in HeLa cell lines showing the mechanism of cellular up take.

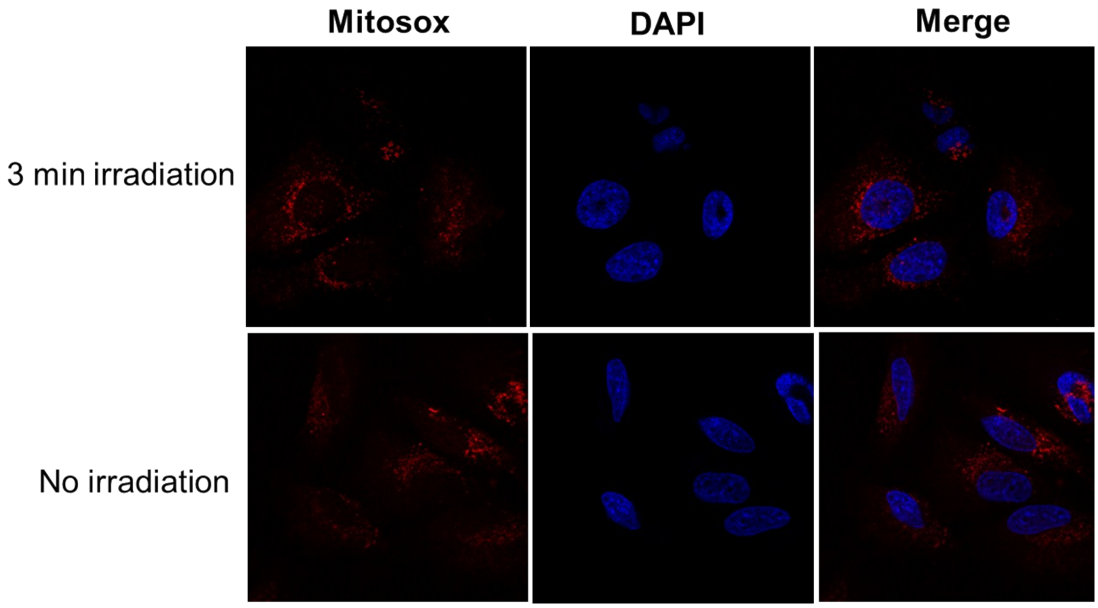

Fig. S22 Confocal images in HeLa cell lines with **IR-780** showing ROS generation. The light irradiation for 3 min was set at 200 mW/cm^2

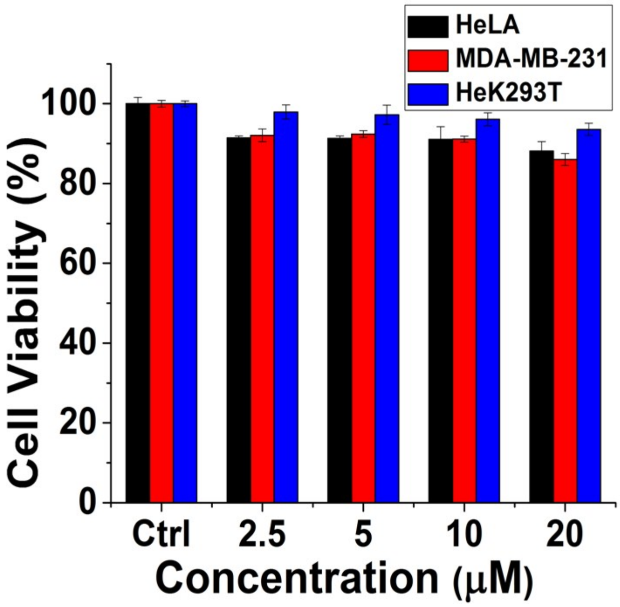

Fig S23. Darktoxicity for HA-Ir-Pyr in HeLa, MDA-MB-231 and HeK293T cells

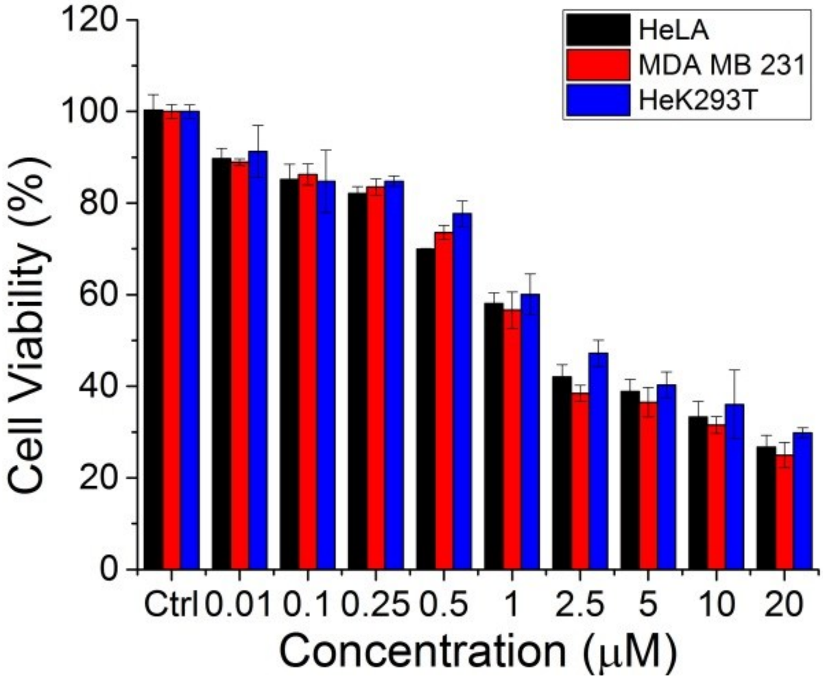

Fig. S24 Dark toxicity of **IR-780**

In vivo experiments

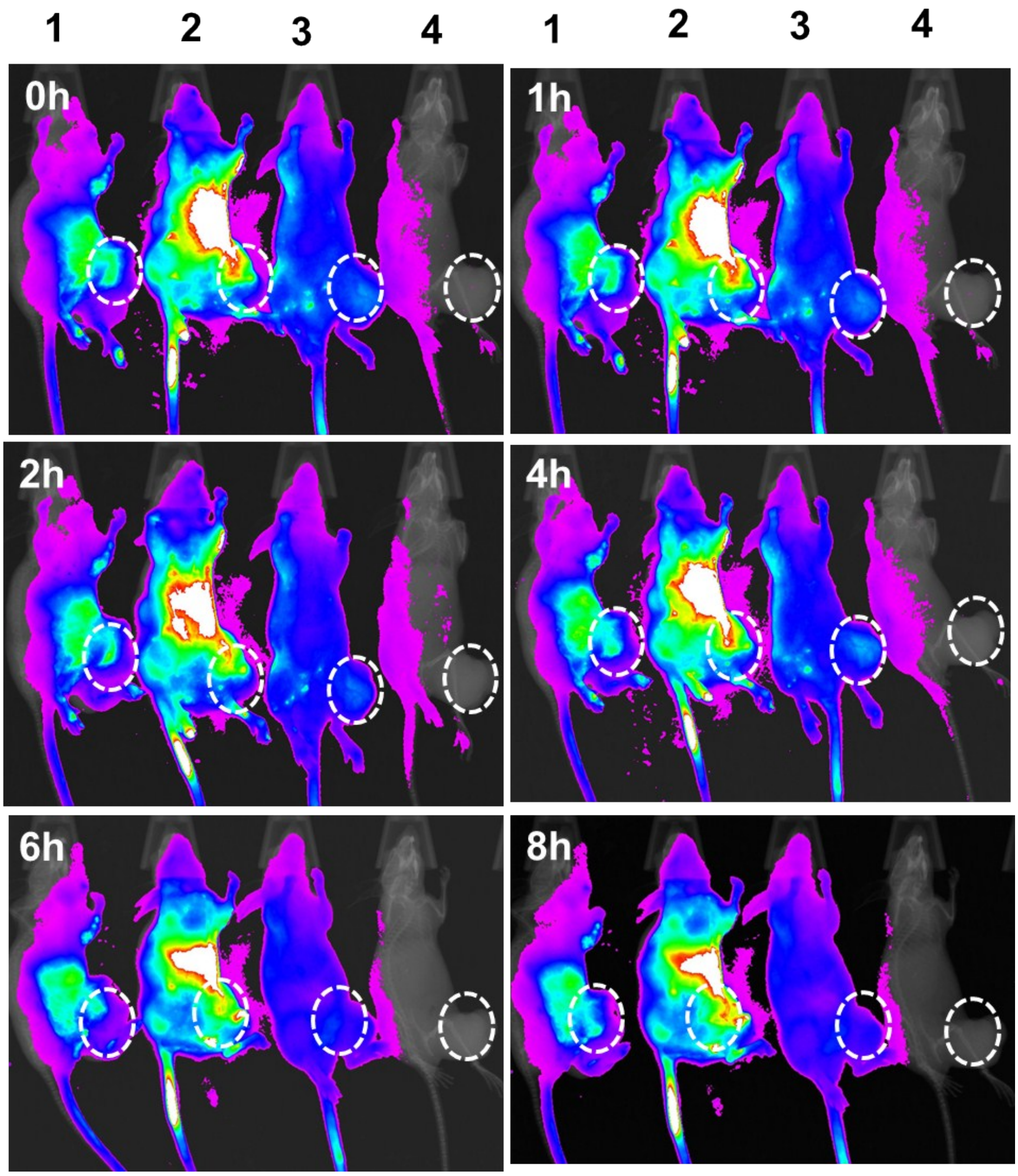

Fig. S25 In vivo imaging in SCC7 xenografted tumor model with, **1**. **IR-Pyr**, **2**. **HA-IR-Pyr**, **3**. **IR-780** and **4**. PBS

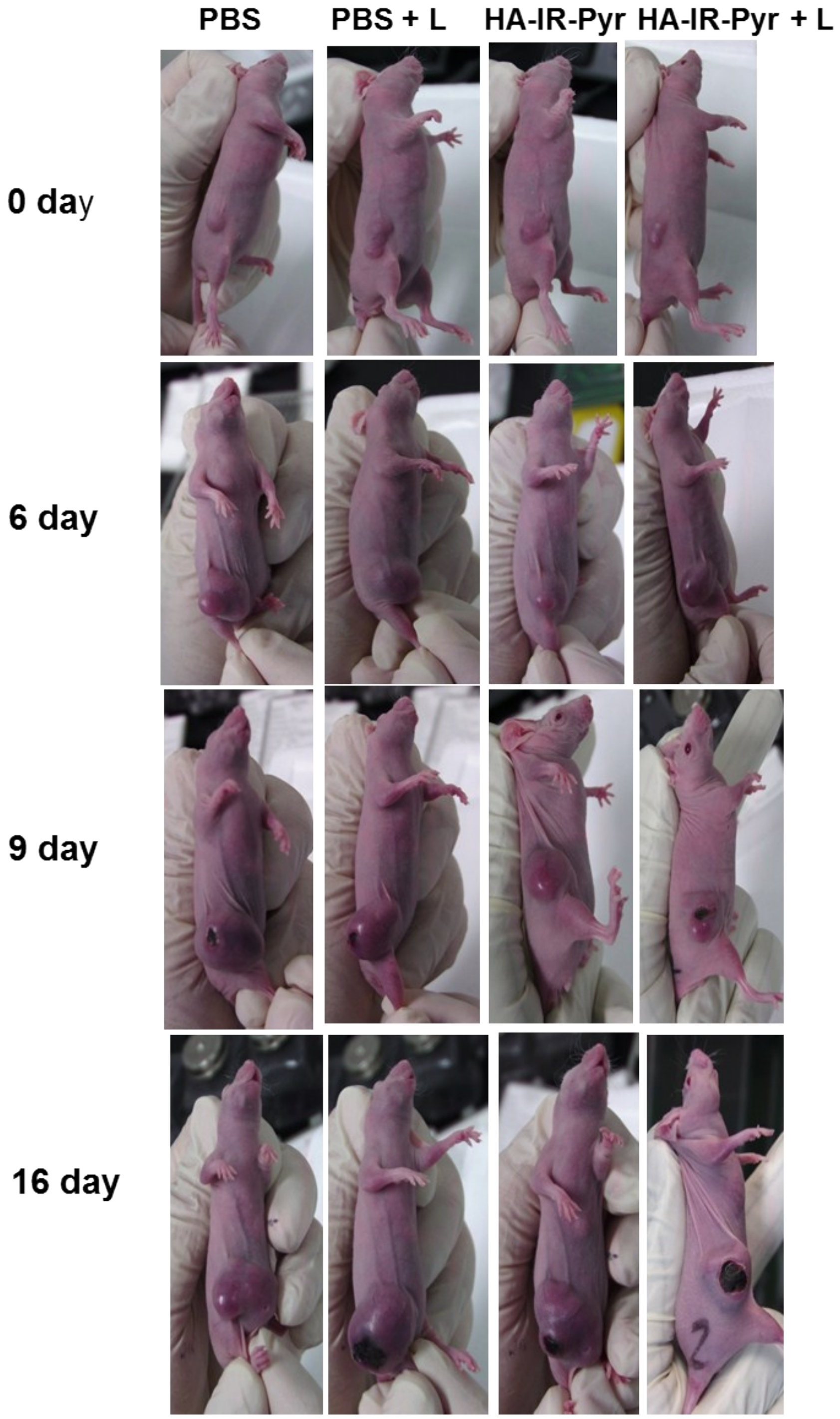

Fig. S26 In vivo experiment showing the PDT efficacy of **HA-IR-Pyr**

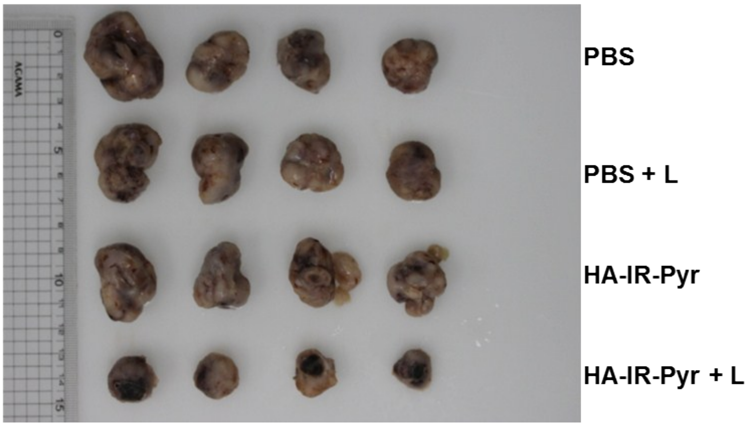

Figure S27. Tumor volume reduction after PDT with HA-IR-Pyr
